# 基于MicroRNAs及其靶基因的化学预防药物对肿瘤抑制的作用

**DOI:** 10.3779/j.issn.1009-3419.2015.04.07

**Published:** 2015-04-20

**Affiliations:** 650118 昆明，昆明医科大学第三附属医院，云南省肿瘤医院肿瘤生物治疗中心 Department of Cancer Biotherapy Center, the Tird Afliated Hospital of Kunming Medical University, Kunming 650118, China

**Keywords:** 化学预防药物, MicroRNAs, 肿瘤, 治疗, 预防, Chemopreventive drugs, MicroRNAs, Tumor, Therapy, Chemoprevention

## Abstract

化学预防药物包括天然化学预防药物和合成化学预防药物，他不仅可预防肿瘤发生，还可在肿瘤治疗中发挥作用。MicroRNAs（miRNAs）是一类短链非编码RNA，通过降解mRNA或抑制mRNA翻译的方式调控着众多基因的表达。近年来越来越多研究表明，在多种肿瘤中化学预防药物通过影响miRNAs及其靶基因的表达发挥对肿瘤的预防和治疗作用，且化学预防药物作用于miRNAs及其靶基因在肿瘤中的实验研究已经展现出良好的安全性和疗效。基于miRNAs及其靶基因的化学预防药物对肿瘤抑制的作用有望成为肿瘤研究新的切入点。本文就天然化学预防药物和合成化学预防药物与miRNAs及其靶基因在肿瘤中的研究进展作一综述。

## Abstract (en)

Chemopreventive drugs including natural chemopreventive drugs and synthetic chemopreventive drugs, it not only can prevent cancer, can also play a role in tumor treatment. MicroRNAs (miRNAs) is a kind of short chains of non-coding RNA, regulating the expression of many genes through the way of degradation of mRNA or inhibitting mRNA translation. In recent years, more and more studies have shown that chemopreventive drugs through influence the expression of miRNAs and their target genes play a role in the prevention and treatment in a variety of tumors, and chemopreventive drugs on the experimental study of miRNAs and their target genes in tumor have demonstrated a good safety and efficacy. Effect on chemopreventive drugs-based microRNAs and their target genes into cancer cells will be expected as a new starting point for cancer research. The thesis expounds and analyzes between the natural chemopreventive drugs and synthetic chemopreventive drugs and miRNAs and their target genes in tumor research progress.

当前，肿瘤是引起全球死亡的头号杀手。中国作为人口最多的国家，正面临着来自肿瘤的严峻挑战^[1]^。尽管近年来手术、放疗和化疗手段，对肿瘤的治疗起到了一定效果，但是肿瘤发病率仍呈现上升趋势，且放化疗的毒副作用、耐药以及肿瘤转移严重阻碍了肿瘤患者总生存率和生活质量。因而加强对肿瘤的预防及寻求安全有效的药物治疗肿瘤引起了人们极大关注。研究显示化学预防药物不仅可用于肿瘤预防^[2, 3]^，也可在肿瘤的治疗上发挥作用^[4, 5]^，但其对肿瘤抑制作用的生物学机制尚未完全清楚，临床上可应用的化学预防药物甚少。因此，阐明化学预防药物对肿瘤抑制的具体生物学机制极为重要。近期大量的研究发现，肿瘤化学预防药物可通过影响miRNAs及其靶基因的表达发挥抗肿瘤活性作用（图 1）。这些研究为阐明化学预防药物对肿瘤抑制的具体生物学机制提供了新的思路，也为临床的应用提供了依据。

**1:**
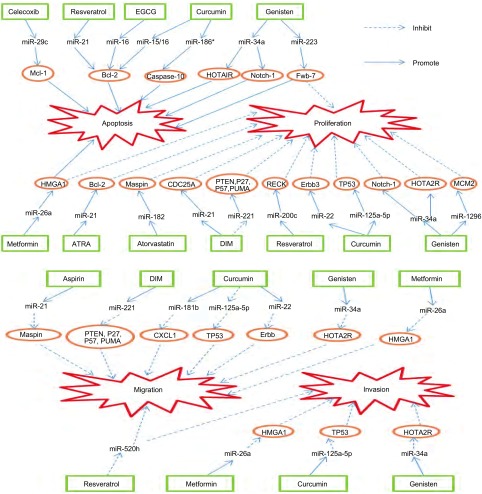
化学预防药物对miRNAs及其靶基因的调控机制 Modulation of miRNAs and their targets by anti-cancer chemopreventive agent. MicroRNAs: miRNAs; DIM: 3, 3-diindolylmethane; EGCG: epigallocatechin gallate; ATRA: all-trans-retinoic-acid.

## 1 化学预防药物

肿瘤化学预防是指利用天然或合成的化学制剂阻止、减缓或逆转肿瘤的发生发展，以达到降低肿瘤的发生率和死亡率的方法^[6]^。化学预防药物一般是指用于肿瘤化学预防的药物，分为天然药物和人工合成药物两大类。到目前为止，已发现可能具有肿瘤预防作用的药物达到数百种，其中这些化合物主要是食物来源的天然化合物，常见如白芦藜醇、吲哚类、姜黄素等；此外还包括一些传统的人工合成药物如非甾体类抗炎药物、他汀类药物等。近年来大量的研究表明，化学预防药物不仅可用于肿瘤预防^[2, 3]^，也可在肿瘤的治疗中发挥作用^[4]^，而其防治肿瘤极为重要作用机制之一是影响miRNAs及其靶基因表达。一方面，化学预防药物影响miRNAs及其靶基因表达抑制肿瘤细胞的增殖^[7]^、诱导凋亡^[8]^、转移^[9]^和增加化疗药物敏感性发挥治疗肿瘤的作用；另一方面，化学预防药物影响miRNAs及其靶基因的表达阻断致癌物活性^[10]^、减轻炎性反应^[11]^及干预癌前疾病^[12]^预防肿瘤发生。

## 2 MicroRNAs（miRNAs）简介

miRNAs是在真核生物中发现的一类内源性的具有调控功能的非编码RNA，其长度约为22个核苷酸。成熟的miRNAs是由较长的初级转录物经过一系列核酸酶的剪切加工而产生，随后组装进RNA诱导的沉默复合体（RNA-induced silencing complex, RISC），通过碱基互补配对的方式识别靶mRNA，并根据互补程度的不同指导沉默复合体降解靶mRNA或者阻遏靶mRNA的翻译^[13]^。目前已明确的人类miRNAs有2, 600多种（miRBase; http://www.mirbase.org/），大量的研究证实，这些miRNAs不仅参与个体发育、细胞凋亡、增殖及分化等正常生命活动^[14-16]^，更在肿瘤发生、发展过程中起到至关重要的作用^[17, 18]^。同时，这些研究也揭示了miRNAs有望成为肿瘤预防和治疗中干预的新靶点。

## 3 天然的化学预防药物与miRNAs

### 3.1 白藜芦醇

白藜芦醇（Resveratrol）是一种从葡萄、花生等水果中提取出来的植物抗毒素，他以不同机制发挥抗肿瘤的活性如诱导DNA损伤^[19]^、调控信号转导^[20]^，而通过调控miRNAs及其靶基因表达对肿瘤抑制是其最为重要的作用方式之一。

#### 3.1.1 白藜芦醇调控miRNAs及其靶基因对肿瘤的治疗作用

研究^[8]^发现，白藜芦醇在PANC-1、CFPAC-1和MIAPaca-2三株胰腺癌细胞中均可通过抑制miR-21靶向调控B淋巴细胞瘤-2基因（B-cell lymphoma-2, BCL-2）诱导肿瘤细胞凋亡。与此相应的是，研究人员在膀胱癌细胞系T24和5637中也发现同样的调控机制^[21]^。另有研究^[22]^发现，白藜芦醇可诱导结肠癌细胞中miR-34a的表达，且miR-34a可靶向下调E2F转录因子3（E2F transcription factor 3, E2F3）及其下游的去乙酰化酶Sirt1（Sirtuin type 1）的表达抑制肿瘤细胞的增殖。此外，在肺癌H460细胞中，白藜芦醇可诱导miR-200c的表达，而上调的miR-200c能靶向抑制*RECK*（reversion-inducing-cysteine-rich protein with kazal motifs）基因的表达，从而激活JNK信号通路和内质网应激，最终抑制肿瘤细胞的增殖^[7]^。最近的一项研究^[9]^表明，在肺癌A549细胞中，白芦藜醇可通过抑制miR-520h介导的信号级联放大效应降低下游的FOXC2表达进而抑制肿瘤细胞迁移和侵袭，并且在肺癌临床样本中得到了相同的结论。可见，白藜芦醇具有显著抗肿瘤活性，在多种肿瘤中显示了它通过影响miRNAs及其靶基因表达进而发挥抗肿瘤的作用，这为白藜芦醇应用于肿瘤的治疗提供了依据。

#### 3.1.2 白藜芦醇调控miRNAs对肿瘤的预防作用

有研究^[10]^利用白芦藜醇抑制雌二醇诱导雌性ACI小鼠成乳腺癌的研究中发现：服用白芦藜醇小鼠的乳腺癌发病率低于对照组，而组织芯片显示：miR-21、miR-129、miR-204和miR-489表达量在肿瘤组织中较正常组织高。同时，检测到与乳腺癌发生密切相关的DNA甲基转移酶3B（DNA methyltransferase 3b, DNMT3b）在肿瘤组织中表达显著低于正常组织。进一步研究证实：白芦藜醇通过影响miR-21、miR-129、miR-204和miR-489与DNMT3b的表达抑制乳腺癌的发生，但DNM3b与上述几个miRNAs的调控关系有待进一步研究。此项研究为乳腺癌的预防提供了新思路。白芦藜醇通过诱导具有抗炎性作用的miR-101b和miR-455表达，显著地抑制右旋糖酐硫酸酯钠（DDS）诱导Apc(Min/+)小鼠结肠炎和息肉发生^[11]^，而结肠炎和息肉被认为是结肠癌的重要病因^[23]^。因此，白芦藜醇可能通过介导miR-101b和miR-455预防结肠癌发生，但寻找与miR-101b和miR-455相应的靶基因有待进一步研究。

综上所述，白藜芦醇通过作用于miRNAs及其靶基因的表达对肿瘤抑制具有巨大潜力，但未来还应开展大量的临床实验进一步验证白芦藜醇对肿瘤抑制作用。

### 3.2 吲哚类

吲哚-3-甲醇（Indole-3-carbinol, I3C）是来源于十字花科蔬菜，在体内代谢产物是3, 3'-二吲哚甲烷（3, 3-diindolylmethane, DIM），I3C和DIM可通过调控细胞周期、细胞增殖和细胞凋亡等发挥肿瘤抑制的作用^[24, 25]^。

#### 3.2.1 吲哚类调控miRNAs及其靶基因对肿瘤的治疗作用

研究人员^[24]^发现，在乳腺癌MCF-7细胞中DIM可增加miR-21的表达，重要的是，上调的miR-21通过降解细胞分裂周期25同源物A（cell division cycle 25 homolog A, CDC25A）的表达进而抑制肿瘤细胞的增殖。而在胰腺癌中研究发现，BR-DIM（生物合成的DIM）可通过抑制miR-221表达来减少对人第10号染色体缺失的磷酸酶及张力蛋白同源的基因（phosphatase and tensin homolog deleted on chromosome ten, PTEN）和调控细胞周期进程的p27(kip1)、p57(kip2)基因以及p53上调凋亡调控因子（p53 up-regulated modulator of apoptosis, PUMA）基因作用，从而抑制肿瘤细胞的增殖和迁移^[25]^。值得关注的是，Paik等^[26]^研究显示，I3C可通过调控miR-21的表达增加吉西他滨对胰腺癌的化疗敏感性，大大提高了吉西他滨的疗效。

#### 3.2.2 吲哚类调控miRNAs及其靶基因对肿瘤的预防作用

Melkamu等^[27]^用I3C抑制乙烯基氨基甲酸酯诱导A/J系小鼠成瘤过程研究结果显示：服用乙烯基氨基甲酸酯加I3C的小鼠成瘤率低于仅服用乙烯基氨基甲酸酯的对照组，而miRNA芯片显示：实验中miR-21、miR-31、miR-130a、miR-146b和miR-377的表达显著低于对照组，但都高于正常组织。进一步研究发现，miR-21可靶向降解程序性细胞死亡4（programmed cell death 4, PDCD4）进而诱导细胞增殖。因此，以上研究表明I3C可通过调控癌基因miR-21来防致癌物的成瘤作用，而深入研究I3C调控miRNAs及其靶基因的机制将对I3C预防肿瘤的发生具有重要的指导意义。

### 3.3 姜黄素

姜黄素（Curcumin）是姜科姜黄属植物的天然产生的多酚，近期研究^[28-31]^发现，姜黄素在多种肿瘤中可通过影响miRNAs及其靶基因发挥抑制肿瘤细胞增殖、迁移、侵袭和诱导细胞凋亡作用，并且还可增加化疗药物的敏感性。

#### 3.3.1 姜黄素调控miRNAs及其靶基因对肿瘤的治疗作用

在乳腺癌中，姜黄素处理乳腺癌MCF-7细胞后miR-15a和miR-16表达显著上升，且二者共同靶向作用于*Bcl-2*基因进而引起肿瘤细胞凋亡^[28]^。此外，姜黄素在乳腺癌MDA-MB-231细胞系中可上调miR-181b发挥抑制肿瘤细胞增殖和诱导凋亡的作用，进一步研究^[29]^发现，上调的miR-181b还可靶向作用趋化因子CXCL1抑制了肿瘤细胞的迁移。在视网膜母细胞瘤中，姜黄素处理视网膜母细胞瘤细胞能显著上调miR-22表达，而上调miR-22可靶向作用于表皮生长因子受体3（Erb-b2 receptor tyrosine kinase 3, ErbB3）进而抑制肿瘤细胞增殖和迁移^[30]^。在鼻咽癌中，Gao等^[31]^研究发现，姜黄素通过抑制鼻咽癌HONE1细胞中miR-125a-5p靶向作用于*TP53*基因来抑制肿瘤细胞增殖、迁移和侵袭。以上的研究显示了姜黄素对肿瘤的抑制作用潜力巨大。

值得关注的是，姜黄素还可通过调控miRNAs的表达增加化疗药物的疗效。研究^[32]^发现用姜黄素处理肺癌A549细胞系，miR-186^*^表达显著下调，进一步研究证实，miR-186^*^是通过靶向调控caspase家族蛋白酶Caspase-10诱导肺癌细胞凋亡。令人感兴趣的是，相同的结论在人肺腺癌耐药细胞株A549/DDP也得以验证^[33]^。另一项研究中，Noratto等^[34]^证实在结肠癌HT-29和SW-480细胞中，类姜黄素可通过诱导miR-27a-ZBTB10-Sp信号轴增加5-氟尿嘧啶的化疗敏感性，大大提高了5-氟尿嘧啶对结肠癌细胞的杀伤作用。这些研究为改善化疗药物的耐药提供了新的思路。

#### 3.3.2 姜黄素调控miRNAs及其靶基因对肿瘤的预防作用

Hassan等^[12]^研究发现，姜黄素可通过抑制四氯化碳（CCL4）诱导小鼠肝纤维化及肝硬化，进一步研究发现，miR-199和miR-200在这此过程中发挥着关键作用。而另一项研究中，姜黄素也可通过上调miR-29b靶向作用DNA甲基转移酶3b（DNA methyltransferase 3b, DNMT3b）降低*PTEN*基因的DNA甲基化水平进而抑制肝纤维化及肝硬化^[35]^。重要的是，肝硬化属于肝癌的高危因素^[36]^，这一定程度说明姜黄素可预防肝癌的发生。

姜黄素对肿瘤的防治有着巨大潜力，但在体内的低生物活性限制了它的抗肿瘤效果。因此，对于如何提高姜黄素的体内活性有待于进一步研究。

### 3.4 染料木黃酮

染料木黄酮（Genistein）是一种大量存于大豆中并已被广泛研究具有化学预防作用的异黄酮^[37]^。大量的研究显示了染料木黄酮在前列腺癌^[38]^、肾癌^[39]^和胰腺癌^[40]^中可通过miRNAs及其靶基因抑制细胞增殖、侵袭、迁移和诱导细胞凋亡。

#### 3.4.1 姜黄素调控miRNAs及其靶基因对肿瘤的治疗作用

在前列腺癌中，研究证实染料木黄酮可调控miR-1296、miR-34a和miR-1260b的表达来影响肿瘤细胞的增殖。Majid等^[38]^发现，染料木黄酮在前列腺癌PC-3细胞系中可诱导miR-1296的表达，且上调的miR-1296可通过抑制微小染色体维持蛋白2（minichromosome maintenance complex component 2, MCM2）的表达抑制肿瘤细胞增殖。而Xia等^[41]^研究发现，染料木黄酮能诱导前列腺细胞中miR-34a的表达，并靶向下调Notch-1基因抑制增殖并诱导细胞凋亡。另有研究^[42]^发现，染料木黄酮可上调的前列腺癌PC3和DU145细胞中miR-34a的表达，重要的是，miR-34a可下调HOX转录反义RNA（HOX transcript antisense RNA, HOTAIR）而抑制细胞增殖、迁移、侵袭和诱导凋亡。新近研究^[43]^显示，染料木黄酮还可下调前列腺癌细胞中miR-1260b的表达抑制肿瘤细胞增殖和侵袭。可见，染料木黄酮可通过对miR-1296、miR-34a、miR-1260b及其靶基因的调控对前列腺癌起治疗作用。

除了前列腺癌，染料木黄酮还在其他多种恶性肿瘤中调控miRNAs及其靶基因发挥治疗的作用。在肾癌中，染料木黄酮通过调控miR-1260b表达抑制Wnt信号通路进而抑制细胞增殖，侵袭并促细胞凋亡^[39]^。在胰腺癌中，染料木黄酮通过抑制miR-223的表达上调泛素连接酶FBW7进而抑制细胞增殖和诱导细胞凋亡^[40]^。在另一项研究^[44]^中，染料木黄酮可通过调控miR-27a的表达抑制细胞的增殖、迁移并诱导细胞凋亡。

### 3.5 表没食子儿茶素没食子酸酯

表没食子儿茶素没食子酸酯（epigallocatechin gallate, EGCG）是来自绿茶的多酚，研究证实其在人类多种肿瘤中具有抗癌的特性。

EGCG调控miRNAs及其靶基对肿瘤的治疗作用：在人恶性神经母细胞瘤中，Chakrabarti研究小组^[45, 46]^证实，EGCG可引起恶性神经母细胞瘤SH-SY5Y和SK-N-DZ细胞中miR-92、miR-93和miR-106b的下调，同时也上调miRNAs（miR-7-1、miR-34a和miR-99a），进一步研究发现，上调的miR-7-1可显著诱导神经母细胞瘤细胞的凋亡。在肝癌中，研究^[47]^发现EGCG可诱导肝癌HepG2细胞中miR-16的表达，而上调的miR-16可靶向抑制Bcl-2基因进而诱导肝癌细胞的凋亡。在肺癌中，EGCG可通过介导肺癌H1299和H460细胞中缺氧诱导因子1-α（hypoxia inducible factor 1 alpha, HIF1α）诱导抑癌作用miR-210的表达来抑制肿瘤细胞增殖^[48]^。以上研究表明，EGCG主要是通过影响miR-7-1、miR-16和miR-210表达发挥其抗肿瘤活性。

此外，EGCG也能影响miRNA的表达提高化疗药物的敏感性。研究^[49]^发现在肺癌A549细胞系中，EGCG可通过抑制miR-98-5p来提高化疗药顺铂的敏感性和提高抑癌基因p53的表达发挥抗肿瘤作用。未来可用EGCG和顺铂联合治疗肺癌，这样可减少肺癌患者顺铂耐药的程度。

### 3.6 其他天然化学预防药物

除了以上介绍的几种天然化学预防药物可通过影响miRNAs及其靶基因发挥抗肿瘤作用外，还有一些天然化学预防药物也具有相同功能。例如，维生素D（Vitamin D）是一种应用广泛的肿瘤化学预防试剂。研究发现，1, 25-二羟基维生素D3可诱导前列腺癌细胞中miR-98的表达，上调的miR-98可介导G_2_/M期阻滞抑制肿瘤细胞的生长；同时也可靶向作用Cyclin-J影响细胞有丝分裂^[50]^。全反式维甲酸（all-trans-retinoic-acid, ATRA）是动物体内维生素A的代谢中间产物，它可抑制肿瘤细胞增殖和诱导肿瘤细胞分化。在乳腺癌中，ATRA被报道在MCF-7细胞系中可诱导miR-21的表达，而上调的miR-21可靶向作用人乳腺丝抑蛋白（mammary serine protease inhibitor, maspin）进而抑制肿瘤细胞的增殖和迁移^[51]^。以上的天然化学预防药物通过调控miRNAs及其靶基因发挥对肿瘤治疗的作用，相信随着对天然化学药物与miRNAs相关性研究的进展，未来会有越来越多的证据证实天然药物成分对肿瘤抑制的有效性。

## 4 合成化学预防药物与miRNAs

### 4.1 塞来昔布

塞来昔布（Celecoxib）是全球首个选择性非甾体抗炎镇痛药，具有抗炎、镇痛等作用。近期研究^[52]^发现，塞来昔布处理结肠癌HT-29细胞系可抑制肿瘤细胞增殖，进一步研究发现，这与28个异常表达的miRNAs如显著上调的miR-552、miR-96等，和显著下调的miR-145^*^、miR-571等密切相关。Saito等^[53]^在胃癌中研究证实，miR-29c在胃癌组织中表达低于正常的胃粘膜组织，而服用塞来昔布miR-29c显著表达，进一步研究发现，miR-29c靶向抑制癌基因*Mcl-1*（myeloid cell leukemia^-1^）的表达进而诱导癌细胞的凋亡。以上结果表明塞来昔布可作用miRNAs发挥抗肿瘤特性，今后更多的基础和临床研究可能利用塞来昔布治疗肿瘤。

### 4.2 硫化舒林酸

硫化舒林酸（Sulindac sulfide）属于NSAIDs的一员，大量研究^[54-56]^显示，NSAIDs中舒林酸硫化物具有抑制肿瘤细胞增殖和诱导细胞凋亡作用，但很少报道它能抑制肿瘤转移的作用。而近期的一项研究^[57]^显示，硫化舒林酸在结肠癌HCT116细胞中可通过抑制核转录因子kappa B（nuclear factor of kappa B, NF-κB）来影响4个已证实具有抑制肿瘤侵袭的miRNAs（miR-10b、miR-17、miR-21和miR-9）的表达进而抑制肿瘤细胞的转移。以上的研究提示，miRNAs在功能上参与舒林酸硫化物的抗侵袭和转移活性，但其具体调控的靶基因有待进一步研究。因此，未来硫化舒林酸可能通过作用于miRNAs来发挥抗肿瘤作用。

### 4.3 阿司匹林

阿司匹林（Aspirin）也是NSAIDs的一种，作为一种可降低结直肠癌发病率及病死率的药物引起人们的关注，但对其具体的机制未完全阐明。研究^[58]^发现，在人骨肉瘤U2OS细胞中，阿司匹林可通过抑制NF-kB的活性来诱导miR-34a过表达进而促进肿瘤细胞凋亡。最近的一项研究^[59]^发现，在结肠癌中发现阿司匹林能抑制miR-21的表达，而下调miR-21可通过激活β-catenin/TCF4信号进而抑制癌细胞的增殖。上述研究说明，阿司匹林通过影响miRNAs及其靶基因表达发挥抗肿瘤作用，也为理解阿司匹林预防肿瘤提供了新思路。

### 4.4 阿伐他汀

阿伐他汀（Atorvastatin）是他汀药物的一种，目前主要用于降低高胆固醇，其所能抑制的3-羟基-3-甲基戊二酰辅酶A还原酶现亦已被认为在肿瘤发展中起着一定作用。研究^[60]^发现，在前列腺癌PC3细胞中，阿伐他汀以一种剂量和时间依赖性的方式抑制肿瘤细胞的增殖，进一步研究发现，阿伐他汀通过诱导miR-182表达和抑制*Bcl-2*基因发挥抑制肿瘤细胞增殖的作用，而*Bcl-2*被鉴定为miR-182潜在的靶基因。此项研究提示了阿伐他汀可影响miR-182及其靶基因*Bcl-2*表达抑制肿瘤增殖。虽然这只是细胞水平的研究，但在一定程度揭示了阿伐他汀具有抗肿瘤的作用。

### 4.5 二甲双胍

二甲双胍（Metformin）是一种用于治疗2型糖尿病的口服降糖药，而近期大量的流行病学研究提示二甲双胍可降低恶性肿瘤的发生和死亡率，其可能机制是通过影响一些抑制肿瘤干细胞效应的miRNAs和致癌功能的miRNAs表达来发挥抗肿瘤作用。研究发现在乳腺癌中，miRNAs如let-7表达下降引起细胞持续进展性的去分化效应（如上皮细胞间质化）使之发生“干细胞化”样改变^[61]^，而肿瘤干细胞对肿瘤的增殖、转移及复发具有重要作用。二甲双胍可通过上调恶变前的细胞let-7的表达可逆转这一效应，可阻断肿瘤细胞的细胞转变及肿瘤干细胞的形成^[62]^。在胰腺癌中，二甲双胍可诱导胰腺癌细胞中部分miRNAs如let-7a、let-7b、miR-26a、miR-101、miR-200b和miR-200c表达，进一步研究^[63]^发现，通过转染使miR-26a的再表达降低了肿瘤干细胞特异性基因的表达水平。此外，二甲双胍存在计量依赖性方式诱导胰腺癌细胞中miR-26a、let-7c、miR-192表达，而上调的miR-26a可通过靶作用高迁移率族蛋白A1（high mobility group protein, HMGA1）抑制细胞增殖、侵袭、迁移和诱导细胞凋亡，并且相同的结论在裸鼠异种移植模型中也得以验证^[64]^。近期研究^[65]^发现，在肺癌A549细胞和NCI-H358细胞中二甲双胍可抑制miR-222的表达，下调的miR-222可减少对靶基因*P27*、*P57*和PTEN的降解，进而抑制肿瘤细胞增殖。可见，二甲双胍通过影响miRNAs的表达发挥抗肿瘤活性。

## 5 结语与展望

化学预防药物与miRNAs是近年来的研究热点。化学预防药物通过影响miRNAs及其作用的靶基因表达发挥的抗肿瘤特性，揭示了化学预防药物对肿瘤抑制的具体生物学作用机制。由于上述化学预防药物在细胞水平和动物水平证实具有较好的抗肿瘤效果，大批化学预防药物在治疗肿瘤的临床试验已经开展。因此，化学预防药物在肿瘤的防治研究工作中具有巨大潜力。综上，化学预防药物通过影响miRNAs及其靶基因表达作为肿瘤防治的新方法可提高肿瘤患者总生存率和生活质量，为肿瘤预防带来了新的希望。
